# The Biosorption of Copper(II) Using a Natural Biofilm Formed on the Stones from the Metro River, Malang City, Indonesia

**DOI:** 10.1155/2022/9975333

**Published:** 2022-09-27

**Authors:** Andi Kurniawan, Siti Mariyah Ulfa, Chamidah Chamidah

**Affiliations:** ^1^Faculty of Fisheries and Marine Science, University of Brawijaya, Malang, Indonesia; ^2^Microbial Resources and Biotechnology Research Group, Graduate School of University of Brawijaya, Malang, Indonesia; ^3^Faculty of Mathematics and Natural Sciences, University of Brawijaya, Malang, Indonesia; ^4^Graduate School of Life Sciences, Ritsumeikan University, Kyoto, Japan

## Abstract

Biofilm is the predominant habitat of microbes in aquatic ecosystems. Microhabitat inside the biofilm matrix is a nutrient-rich environment promoted by the adsorption of nutrient ions from the surrounding water. Biofilms can not only adsorb ions that are nutrients but also other ions, such as heavy metals. The ability of biofilm to attract and retain heavy metals, such as copper(II), makes biofilms a promising biosorbent for water pollution treatment. The present study analyzes the characteristics of copper(II) adsorption by biofilms naturally formed in the river. The biofilms used in this study grow naturally on the stones in the Metro River in Malang City, Indonesia. Methods to analyze the adsorption characteristics of copper(II) by biofilms were kinetics of the adsorption and adsorption isotherm. The maximum adsorption amount and the adsorption equilibrium constant were calculated using a variant of the Langmuir isotherm model. In addition, the presence of the functional groups as suggested binding sites in biofilm polymers was investigated using the Fourier transform infrared (FTIR) analysis. The results indicate that copper(II)'s adsorption to the biofilm is a physicochemical process. The adsorption of copper(II) is fitted well with the Langmuir isotherm model, suggesting that the adsorption of copper(II) to a biofilm is due to the interaction between the adsorption sites on the biofilm and the ions. The biofilm's maximum absorption capacity for copper(II) is calculated to be 2.14 mg/wet-g of biofilm, with the equilibrium rate constant at 0.05 L/mg. Therefore, the biofilms on the stones from river can be a promising biosorbent of copper(II) pollution in aquatic ecosystems.

## 1. Introduction

One of the major environmental problems is the contamination of aquatic ecosystems by various pollutants, such as heavy metals [1–3]. Copper(II) is a widely used in various industries [4–6] and may exist in the water stream from various utility plants, such as metal plating, mining, manufacturing of computer, ceramic glazing, or electricity [7–9]. Copper(II) ions are toxic nondegradable and their increasing concentration in the water present a severe hazard to human health or the environment [10–12]. Thus, it is critically important to reduce copper(II) contamination in aquatic ecosystems.

Biosorption appears as a promising, inexpensive, and environmentally friendly alternative technology for reducing heavy metal contamination in aquatic ecosystems [13–15]. The effectiveness of biosorption is significantly affected by the choice of biosorbent [16]: [17, 18]. The biosorbent should be ubiquitous in aquatic ecosystems and easy to obtain. Therefore, aquatic microbes that exist in large numbers and play various essential function in almost all parts of aquatic ecosystems [19–22] have emerged as a promising candidate for biosorbents.

The predominant habitat of microbes in aquatic ecosystems is a biofilm [23, 24]. Biofilms formed naturally in aquatic ecosystems can be defined as the polymicrobial communities embedded in extracellular polymeric substances and attached on the surface [25, 26]. The biofilm serves various functions in an aquatic ecosystem, such as cycling nutrients and purifying pollutants [27, 28]. A biofilm may accumulate various ions, such as copper(II), from the surrounding water by attracting the ions and retaining them inside the biofilm matrix [29, 30]. Hence, the biofilm may be a promising biosorbent for copper(II) contamination in aquatic ecosystems, including rivers. However, most of the research focuses on laboratory-grown or single-species biofilms. There has been little focus on utilizing natural biofilm formed in the river as a biosorbent of copper(II), especially biofilms from the tropical aquatic ecosystems. The present study investigates the mechanism with which copper(II) adsorbs to biofilms collected from the river in Malang city of Indonesia to examine biofilm's potential in the biosorption of heavy metals pollutants. According the results of this study, a biofilm naturally formed in the river is a promising biosorbent to immobilize copper(II) in aquatic ecosystems including rivers.

## 2. Material and Methods

### 2.1. Sample Preparation

Biofilms formed on the stones were collected from the Metro River in Malang City, Indonesia. Biofilm samples were taken in early September 2019, which is included in the dry season. The Metro River flows through industrial and domestic areas and is thus susceptible to water pollution. The stones were collected from the water that was about 50 cm in depth and were brought back to the laboratory in a plastic container filled with river water. The temperature of the container was maintained at approximately 4 °C. The biofilms were removed from the surface of the stones with a toothbrush and resuspended in distilled water. The pellet of the biofilm was prepared by centrifugation at 8,000×*g* and 4 °C for 5 min. The pellet was washed with distilled water and then centrifuged three times.

### 2.2. Kinetics of Adsorption

The time course of copper(II) adsorption to the biofilm was investigated by measuring the amount of Cu adsorbed to the biofilm after 5, 30, 60, or 120 minutes of incubation. Preliminary research shows that adsorption occurs rapidly where the equilibrium condition is reached within 5 minutes. Hence, the first contact time used in this study was 5 minutes. After that, the contact times used are multiples of 30 and 60 minutes (i.e., 30, 60 minutes, and 120 minutes) to see if the adsorption rate remains stable, indicating that the equilibrium concentration has been reached. Ten mL aliquots of 20 mg/L CuSO_4_ were prepared by dissolving reagent-grade CuSO_4_ solution in distilled water. Afterward, 0.2 mg of the biofilm pellet was added to each CuSO_4_ solution and mixed well using a magnetic stirrer at 125 rpm. After each incubation time of 5, 30, 60, or 120 minutes, the biofilm suspension was centrifuged at 8,000 ×*g* for 30 minutes. The Cu concentration in the supernatant was measured using atomic absorption spectroscopy (Shimadzu AA-6800, Shimadzu Corporation, Japan). The amount of copper(II) adsorbed to the biofilm was calculated by subtracting the copper(II) concentration in the control sample, which was a CuSO_4_ solution without biofilms, from the copper(II) concentration in the supernatant of the biofilm suspension. The experiments were conducted in the water bath with automatic temperature control set at 28 °C. pH at the beginning and end of the experiment was measured (pH Meter LAQUA PH1100–S, Horiba, Japan) and showed that the water pH was relatively unchanged (ca. pH 7.0). Each experiment was repeated three times independently.

### 2.3. Adsorption Isotherm

Ten milliliters of CuSO_4_ aqueous solutions of varying concentrations, 5–230 mg/L, were prepared. Then, 0.2 mg of the biofilm pellet was added to each solution and mixed well using a magnetic stirrer at 125 rpm. After 5 minutes, the biofilm suspensions were centrifuged at 8,000 g for 10 minutes to obtain the supernatants to measure copper(II) concentration and the biofilm pellets for the Fourier transform infrared (FTIR) analysis. The copper(II) concentration was measured using atomic absorption spectroscopy. The accumulation of copper(II) was calculated from the differences between the copper(II) concentration in the supernatant of the biofilm suspension and the control, i.e., the CuSO_4_ solution without the added biofilm. The experiments were conducted in the water bath with automatic temperature control set at 28 °C. pH at the beginning and end of the experiment was measured (pH Meter LAQUA PH1100–S, Horiba, Japan) and showed that the water pH was relatively unchanged (ca. pH 7.0). Each experiment was repeated three times independently.

The maximum adsorption amount and the adsorption equilibrium constant were calculated using a variant of the Langmuir isotherm model as described below:

The equation assumes that a dynamic equilibrium exists between the adsorbed copper(II) (N; mmol/wet-g) and the free copper(II) in a solution whose concentration is at equilibrium (C; mg/L). The adsorption equilibrium constant (b) was defined as the ratio of the adsorption and desorption rates. The value of *b* increases as the adsorption rate exceeds the desorption rate, relatively. The plot of C/N against C yields a straight line with a slope of 1/Nmax and a *y*-axis intercept of 1/(Nmax) *b* for the calculation of Nmax (the maximum amount of adsorbed ion; mmol/wet-g) and *b* [31].

### 2.4. FTIR Analysis

The biofilms subjected to the FTIR spectra analysis included intact biofilms and the biofilms after Cu adsorption. Biofilm pellets were dried at approximately 60 °C until reaching a constant weight. The dry pellet was used in FTIR analysis. An amount of 0.01 Gram of the dry biofilm pellets was mixed with powdered potassium bromide (KBr) and pressed under high pressure. Under pressure, KBr melted and sealed the compound into the matrix. The KBr pellet was measured with a Shimadzu FTIR Spectrometer 84002 (Shimadzu Corporation, Japan).

### 2.5. Data Analysis

In this study, data analysis was carried out using descriptive statistical analysis to observe the pattern of relationships between variables. To provide more comprehensive information, the standard deviation is also displayed along with the mean value in the graph to see the dispersion of the distribution. A small standard deviation value indicates a relatively stable relationship compared to a high standard deviation. Furthermore, data analysis was also carried out by linear regression analysis to explore the linear relationship between variables. This analysis will provide information regarding the direction of the relationship between variables and quantitatively measure how changes in the independent variable will affect the changes of the dependent variable. Those two approaches mentioned above, either descriptive or inferential approaches, were performed using the features in Microsoft Excel due to its simplicity and practical use.

## 3. Result and Discussion

### 3.1. Kinetics of Adsorption

**Figure 1:**
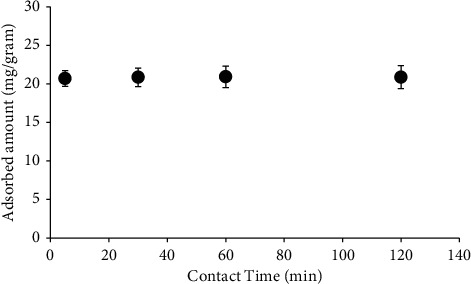
The time course of copper(II) adsorption to the biofilm. The experiment was repeated three times, independently (average values are shown). Bars represent the standard error.

The time course of copper(II) adsorption to the biofilm was investigated. The amount of adsorbed copper(II) to a biofilm remained relatively similar, at approximately 1.0 mg/Gram, from the beginning until the end of the adsorption experiment (Figure 1). The adsorption of copper(II) to biofilm occurred rapidly, where the maximum adsorption amount reached within 5 minutes. Previous studies also reported that the adsorption of ions (i.e., NH_4_^+^ and NO_3_^−^) to biofilm occurred quickly [30]. Rapid adsorption is characteristic of physicochemical adsorption [32]. Thus, the biosorption of copper(II) to biofilms likely occurs through physicochemical mechanisms, such as ion exchange mechanism and electrostatic interaction. Based on the study of the adsorption kinetics, the time course used in the subsequent experiments in this study chose 5 minutes as the contact time.

### 3.2. Adsorption Isotherm

**Figure 2:**
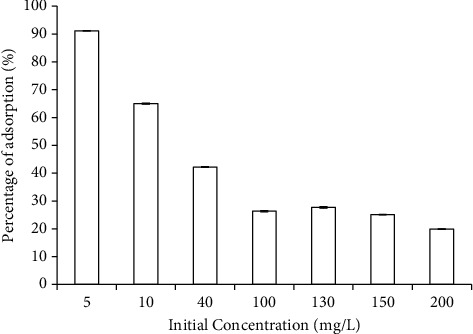
The effectiveness of copper(II) Cu adsorption to the biofilm. The experiment was repeated three times, independently (average values are shown). Bars represent the standard error; however, the bars are not clearly visible due to the small values of standard errors.

The characteristics, such as the adsorption isotherm, of copper(II)'s adsorption to biofilms were investigated in greater detail. First, the amount of copper(II) adsorbed to the biofilm using CuSO_4_ solutions of various initial concentrations was analyzed (Figure 2). The effectiveness of copper(II) adsorption to biofilm decreased, from 91% to 16%, as the initial concentration of the CuSO_4_ solution increased. The high percentage of adsorption attained in low CuSO_4_ concentration seemed to be promoted by the higher ratio of the available adsorption sites in biofilms to the amount of free copper(II) in the surrounding water. When this ratio became smaller, the percentage of copper(II) accumulated in the biofilm also decreased.

**Figure 3:**
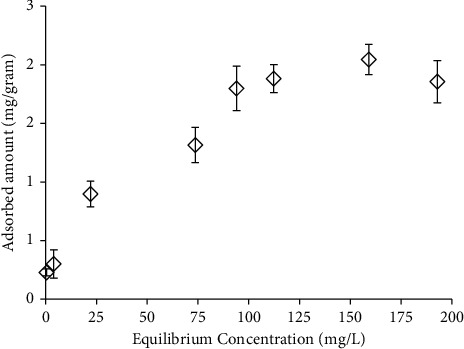
The adsorption isotherm of copper(II) to the biofilm. The experiment was repeated three times, independently (average values are shown). Bars represent the standard error.

The adsorption isotherm of copper(II) to biofilm was plotted (Figure 3). In this graph, the amount of adsorbed copper(II) in different equilibrium concentrations, i.e., the concentration after the adsorption reached equilibrium, was compared. The amount of copper(II) adsorbed to biofilms increased with the increase of equilibrium concentration and then plateaued at high concentrations (>100 mg/L). When equilibrium was reached at low concentrations, not all the adsorption sites on the biofilm bound to copper(II); therefore, the amount of adsorbed copper(II) increased with the increasing concentration of CuSO_4_. On the other hand, all the adsorption sites in the biofilm were already occupied with copper(II) ions at high concentrations of CuSO_4_. Hence, the amounts of copper(II) adsorbed to the biofilms tended to remain stable even though the equilibrium concentration of copper(II) was increased.

**Figure 4:**
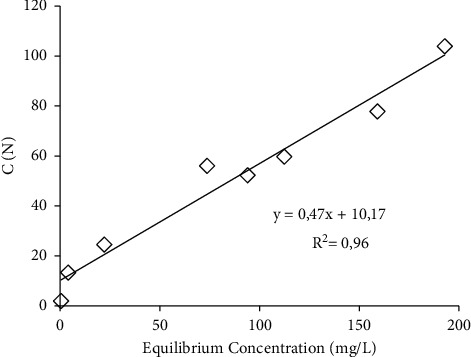
Copper(II) adsorption based on the Langmuir isotherm model.

The adsorption of copper(II) to the biofilm at different equilibrium concentration based on the Langmuir adsorption model was plotted as the ratio of equilibrium concentration to adsorption amount (C/N) against the equilibrium concentration (C) (Figure 4). The result indicated that the adsorption of copper(II) to the biofilm was fitted well with the Langmuir isotherm model (*R*^2^ = 0.96). The adsorption of copper(II) to biofilm seems to occur in monolayer form and is driven by the interaction between the ions and the adsorption sites on the biofilm polymers.

Based on the copper(II) adsorption data plotted to the Langmuir isotherm model, the Nmax and *b* of copper(II) adsorption to biofilm were calculated. The Nmax of copper(II) adsorption to biofilm was estimated to be around 2.14 mg/wet-g, and *b* was at approximately 0.05 L/mg. The accumulation of ions, such as heavy metal, to biofilm, is reportedly due to ion exchange mechanisms and electrostatic interaction [33]. The adsorption sites for the copper(II) are charged sites in biofilm polymers. The charges exist because of the ionization of functional groups in biofilm polymers. Therefore, the functional groups in the biofilm polymer were analyzed to confirm their role in copper(II) adsorption.

### 3.3. FTIR Analysis

The binding sites in biofilm, which can attract ions and retain them inside the biofilm matrix, are ionizable functional groups in the biofilm polymers [34]. Therefore, the presence of the functional groups in biofilm polymers was investigated using FTIR analysis. The biofilm samples included an intact biofilm before adsorption of copper(II) and biofilms with adsorbed copper(II) after incubation with CuSO_4_ solutions of various initial concentrations at 5, 15, 130, and 230 mg/L.

**Figure 5:**
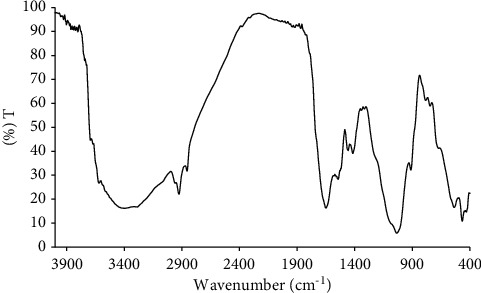
The FTIR spectra of the intact biofilm.

The FTIR spectra of the intact biofilm had a peak at 1653 cm^−1^, indicating the presence of carboxylic groups (*C*=*O*) on the biofilm polymer (Figure 5). The broad spectrum of hydroxyl groups (-OH) at 3600−3100 cm^−1^ supported with the stretching of *C*=*O* indicated the presence of a carboxylic skeleton in the biofilm. The primary amino (-NH) stretching as a weak signal at 3400–3200 cm^−1^ was not detected due to the overlapping with the-OH group. However, the C-N bending was found at 1414 cm^−1^, indicating an amino or amide framework. The presence of a simple aliphatic carboxylic group (-COO-) was detected as sharp peaks at 1730−1700 cm^−1^. The presence of *C*=*C* at 1541 cm^−1^ supported the blueshift of *C*=*O* into 1650 cm^−1^, indicating the conjugated carbonyl framework. The sharp peak at 2926 cm^−1^ was characteristic of the C-H from the methyl (-CH3) or methylene (-CH_2_-) group. The strong and broad band in 1300−1000 cm^−1^ could be attributed to the C-O from the ester group.

**Figure 6:**
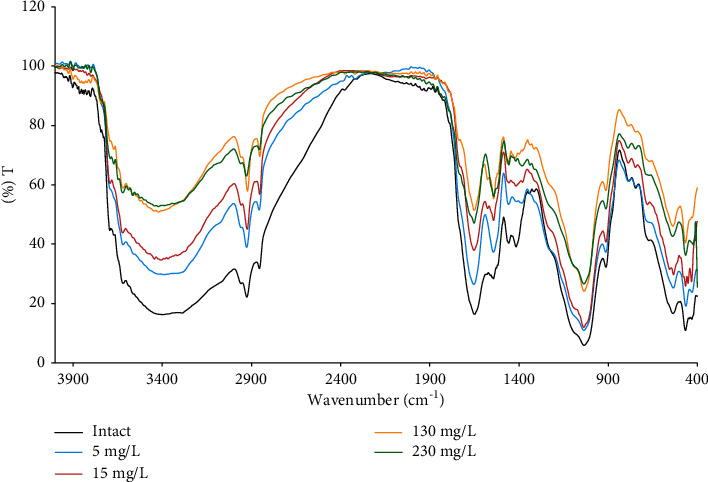
The comparison between the FTIR spectra of intact biofilm and the copper(II)-bound biofilms. The concentrations (mg/L) were the initial concentration of the CuSO_4_ solutions used in the adsorption isotherm experiment.

The FTIR spectra of biofilm were expected to change after the adsorption of copper(II), particularly in the peaks corresponding to the functional groups that acted as binding sites. The FTIR spectra of the copper-bound biofilms were compared to that of the intact biofilm (Figure 6). The considerable decrease in peak intensity at 3410 and 1653 cm^−1^ after the adsorption of copper indicated that the active binding site on the biofilm for copper were the-OH and *C*=*O* groups. The presence of a carboxyl group from the carboxylic acid in the biofilm polymers seemed essential for the copper's adsorption to the biofilm. The -COO- ester groups may contribute to the adsorption of copper moderately since the change in intensity of the peaks around 1042 cm^−1^ was not significant. The adsorption of copper to the biofilm was likely promoted by the interaction between the functional groups and the copper ion. Carboxylic groups have been reported to perform an essential factor in the adsorption of various ions to the biofilm matrix [33]. Therefore, the shifts of the peaks of the functional groups after the copper adsorption were mostly represented by the decreasing peak intensity of the carboxyl groups.

## 4. Conclusion

The present study revealed the following: (1) the adsorption of copper(II) to the biofilm formed naturally on the stones from the river occurs through a physicochemical process promoted by the interaction between the adsorption sites on the biofilm matrices and copper(II); (2) the adsorption sites seem to be the functional groups in the biofilm polymer such as carboxyl and amino groups; (3) the adsorption of copper(II) to biofilm occurs rapidly, reaching the equilibrium state within 5 minutes; (4) the maximum adsorption amount of copper(II) to biofilm is estimated to be 2.41 mg/g, and the equilibrium adsorption constant is estimated to be 0.05 L/mg. According to the result of this study, the naturally formed biofilm on the stones from is a promising biosorbent for treating copper(II) pollution in the river ecosystem.

## Data Availability

The data used to support the findings of this study are included within the article.
